# The influence of distant surcharge load with a finite length on the cantilever walls

**DOI:** 10.1371/journal.pone.0295442

**Published:** 2023-12-11

**Authors:** Recep Akan

**Affiliations:** Department of Civil Engineering, Faculty of Engineering, Suleyman Demirel University, Isparta, Türkiye; University of Duhok, IRAQ

## Abstract

The behavior of a sheet pile wall constructed on saturated sand soil and exposed to a distant surcharge load with a finite length at the top of the backfill soil is examined in this study. For this aim, various internal friction angles (φ), and natural ground surface for the groundwater level are considered. Furthermore, it is considered that the sheet pile wall acts as cantilevered and supports a six-meter-high (*H*) excavation. The simple “*45*° distribution” (*AP*) and uniform distribution of “Beton Kalender distribution” (*BK*) methods are examined with Coulomb’s and Rankine’s earth pressure theories in analytical solutions, while the finite element method (*FEM*) is used as a numerical method. The present research has two primary goals: a) determining the best analytical approach that provides the maximum bending moment (*Mmax*) values that are more comparable to those of the *FEM* b) examining the behavior of the sheet pile wall considering several effects of load scenarios, depth (*D*) and section type (*ST*) of the wall, and the soil properties together. In this context, parametrical analyses are performed. Consequently, it is found that the distance of the surcharge load (*x*_*1*_) has a pronounced effect than the intensity (*q)* and length (*Ls*) of the surcharge load on the behavior of the sheet pile, and this effect vanishes for the large values of *x*_*1*_. Furthermore, Coulomb theory provides more convenient values with *FEM* for *Mmax* than those obtained from Rankine theory. The *Mmax* values obtained from *FEM* are generally less than those from *BK*, while they are greater than those from *APC*.

## Introduction

Sheet pile walls are retaining walls with the advantages of being quick to build, lightweight, resistant to driving pressure, and having a longer service life for situations above and below the water level [1].

Sheet piles can be cantilevered or anchored, depending on the height and properties of the soil, to be retained against lateral forces. Cantilever sheet pile walls are often used to build marine constructions, and they frequently encounter strip surcharge loads from vehicles or building construction behind them [2]. The surcharge load behind the walls increases the lateral pressures, maximum bending moment, and lateral deflection along the sheet pile walls. In the design of sheet pile walls, the maximum moment is one of the most important factors to be considered, especially if they built nearby constructions. Hereby, the tolerable displacement of the walls depends on the soil properties, the location, the significance and the type of nearby structures [3]. As a result, understanding how the surcharge load influences the bending moment and displacement of sheet pile walls is critical for the structural design of sheet pile walls.

The accurate computation of horizontal earth pressures is challenging in designing the sheet pile walls. Therefore, numerous studies have been presented on this subject. The most prevalent approaches for determining active earth pressure without strip load are Coulomb’s and Rankine’s earth pressure theories [2, 4–7]. Coulomb [8] initially offered a way for analytically obtaining the solution to the earth pressure issue by examining the failure wedge and applying the force equilibrium conditions. Rankine [9] proposed an alternative idea identical to Coulomb’s theory but evolved in terms of stress. Additionally, conventional design approaches are even used by Terzaghi [10], Jumikis [11], and Bowles [4], but these approaches include some drawbacks because of different assumptions and concerns [12]. Fang et al. [13] investigated the horizontal pressure caused by a strip loading on a non-yielding wall and drew some conclusions based on the experimental findings. Farzaneh et al. [14] offered a two-dimensional solution using the upper-bound theorem for the combined active earth pressure caused by soil weight and strip foundation surcharge. However, the situation in which backfill is loaded by an infinite strip surcharge load has rarely been investigated.

The elastic analysis [15–17], which mainly relies on Boussinesq’s [18] elastic theory, the simple "*45*° load distribution" [19], the conventional earth pressure analysis of Coulomb [8], and the approach described in Beton Kalender [20] are currently used to determine strip load-generated lateral earth pressures. These methods produce vastly diverse earth pressure distributions, which could result in either excessively conservative or unsafe solutions. Therefore, the significance of this issue and the absence of an appropriate solution have led to an increased amount of study in this field. Motta [21] developed a closed-form approach for walls with sloping fill material and variable surcharge distances. Georgiadis and Anagnostopoulos [22] performed the model experiments of sheet pile walls in the sand to explore the influence of strip loads on the behavior of walls. Ghanbari and Taheri [23] employed the horizontal slices approach and proposed a thorough formulation to estimate the effect of a line surcharge on reinforced retaining walls with frictional or cohesive-frictional backfills. Hatem et al. [24] experimented to assess the influence of surcharge intensity, sloped terrain, and relative soil density on the deflection and bending moment of sheet piles. Xiao and Xia [25] presented a variational calculus technique to compute the passive earth pressure on the rigid retaining with a distant strip surcharge on its top surface about the limit equilibrium conditions of soil mass retained by rigid retaining walls. El-Emam and Touqan [26] created a strip footing next to the non-bending basement wall, where a series of scaled-down models were used to evaluate the basement wall’s performance. Mirmoazen et al. [27] employed the lower-bound limit analysis in conjunction with the finite element discretization method and second-order cone programming to evaluate the active lateral earth pressure on geosynthetic-reinforced retaining walls subjected to overlying strip footing loadings.

Strain, deflections, stress, and bending moments are some of the factors that make geotechnical correlation solutions more complicated. Consequently, numerical modeling has become an essential solution to simplify complicated calculations and improve accuracy [4, 11–14, 28–33]. Nowadays, FEM-based computer software is frequently utilized to provide more realistic designs findings and to understand sheet pile wall behavior better. Using a variety of analytical techniques, Denver and Kellezi [34] tackled several problems and compared them with FEM applied to several typical load scenarios on free and anchored sheet piles. Using three-dimensional FE models, Zhang and Sun [35] investigated the response of piles close to surcharge loads. Rauf et al. [36] presented the deflection of the sheet pile owing to surcharge load on backfill utilizing experimental investigation and numerical analysis using *FEM*-based software. The stability and behavior of the rigid cantilever retaining wall that supports dry sandy soil were investigated by Al-khafaji et al. [37] regarding the location of the surcharge load using a 2D finite element model. Nandi and Choudhury [38] introduced an analytical approach for the displacement-controlled analysis of rigid embedded cantilever retaining walls with a uniform strip surcharge. Later, they compared the settlements to those obtained from the Plaxis 2D program based on FEM. Debnath and Pal [39] applied a two-dimensional nonlinear to investigate the behavior of sheet piles in sand under a uniform surcharge strip foundation load. Then, they compared the deflection and bending moments of the wall to those obtained from Plaxis 2D program based on FEM. Besides, some researchers analyzed the behavior of the sheet pile walls by using the FLAC computer program based on the finite difference method. Singh and Chatterjee [40–42] explored the role of uniform surcharge load on the soil surface at a distance away on the behavior of cantilever sheet pile walls for bending moment, lateral earth pressure, deflection, and settlement behavior. Additionally, Singh and Chatterjee [43] compared the results obtained from Coulomb’s earth pressure theory and the finite difference method to investigate the effect of distant uniform strip load.

As it is seen from the above-mentioned literature research, numerous studies have been conducted on the bending and deflection behavior of sheet pile walls adopting several methods. However, the number of studies on the influence of the location and length of a surcharge load on the sheet pile wall is still very limited. Furthermore, the combined effects of several parameters, i.e., *x*_*1*_, *Ls* and *q*, *φ*, *D*, *ST*, and reduction coefficient of the wall-soil interface (*R*) on the behavior of sheet pile wall, have not been examined yet. In this research paper, an attempt is made to address this problem. Furthermore, some benchmark results for the behavior of the sheet pile walls are provided. In this context, *FEM* analyses are conducted to demonstrate the effects of the relevant parameters on *Mmax* and maximum wall deformation (*Ux*) of the sheet pile wall. Furthermore, four distinct analytical methods are used to solve the *Mmax* of the models combining Coulomb’s and Rankine’s earth pressure theories [8, 9], and *AP* and *BK* distribution approaches [19, 20] to allow one to compare the consistency of widely used analytical solutions with FEM. Consequently, the results are tabulated and illustrated, discussed in detail, and summarized.

## Model

**Fig 1:**
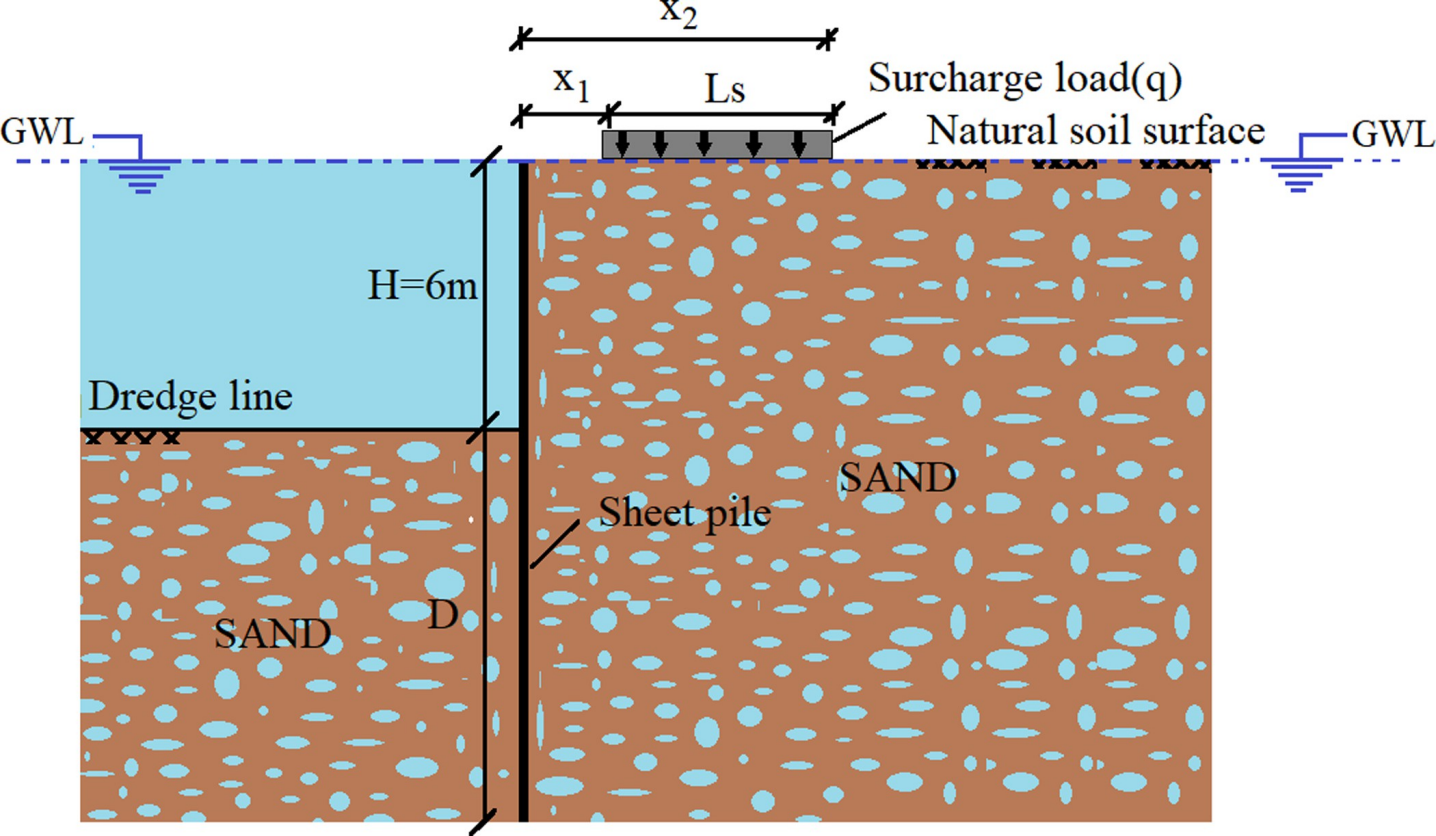
The geometry of the study.

To examine the behaviors of sheet pile walls, not only 400 models are solved with analytical methods, but also 700 models are analyzed with *FEM*. In those models, the groundwater level is assumed to be at the natural soil surface on both sides of the wall, and the saturated unit weight of the soil *γ*_*s*_ = 21 kN/m^3^. The dilatancy angle (*ψ*) of the soil is assumed to be 30 degrees less than *φ*. Then, various analytical and numerical models are developed to examine the effects of the soil, sheet pile, and surcharge load parameters. The parameters considered in the scope of the study and a schematic representation of the relevant model are shown in Table 1 and Fig 1, respectively.

**Table 1:** The parameters of the study.

Parameter	Unit	Values
*φ*	^ο^	25, 30, 35, 40, 45
*R*	-	0.33, 0.67, 1
*ST*	-	PZ22, PZ40
*D*	m	9, 12, 15
*x*_*1*_	m	0, 0.5, 1, 2, 4, 6, 12
*Ls*	m	0, 1, 2, 4, 6, 12, 21, *∞*
*q*	kN/m/m	0, 15, 50

## Method

The analytical solutions focus on the beginning and end of the construction, while the FEM considers state changes caused by deformation in progress. Moreover, the FEM uses the matrix and determinant methods and creates approximate solutions, but analytical methods provide conclusive results. Therefore, analytical methods may provide different results from the FEM results. Since analytical methods are frequently used to solve problems when the FEM is not an option, it is helpful to understand how similar the results of analytical solutions are to the results obtained with the FEM. Hence, both analytical solutions and numerical analyses are presented in the current study, where the Matlab R2015a program is utilized to code the analytical solutions, while the Plaxis 2D V20 is used to perform the *FEM* analyses. It should be noted that in the analytical solutions, the earth pressure theories of Coulomb [8] and Rankine [9] are taken into account together with the load distribution methods of simple “*45*° distribution”(*AP*) [19] and uniform distribution form of "Beton Kalender distribution" (*BK*) [20]. In this context, four different analytical solutions are obtained, considering Coulomb’s earth pressure theory with *AP* and *BK*, and Rankine’s earth pressure theory with *AP* and *BK*, abbreviated as *APC*, *BKC*, *APR* and *BKR*, respectively.

### FEM

The FEM is one of the several numerical methods that have been continuously developed and improved over the last 50 years for evaluating sheet piles [44]. Since the FEM breaks down problems into several components and generates solutions that include derivatives and integrals using matrix and determinant methods. Therefore, it is recognized as a numerical method even if it incorporates analytical approaches to studies because it reveals approximate findings [30]. The *FEM*-based computer software Plaxis 2D v20 which assumes a two-dimensional plane strain and axisymmetric models and lets to perform deformation, stability, and flow analysis for various geotechnical applications. Furthermore, in the related software, the finite element mesh and geometry model can be easily generated [45]. The following assumptions are considered in the numerical analyses:

- The width and length of the models are considered eight times the sheet pile length behind the wall and five times the sheet pile length below the excavation level, respectively, to avoid considerable fluctuation in the findings [46].
- The boundary conditions are considered to be free vertically and constrained horizontally for the vertical borders while fully fixed for the lower horizontal border.
- In accordance with the open literature [28, 30, 34, 39–43, 47–56], sand is modeled in drained conditions using the Mohr-Coulomb constitutive model, while the mesh is generated in the size of “fine” using 15-noded triangular finite elements.
- Elastic plate elements are used to define the sheet piles, where the interface elements are assigned on both sides of the wall to provide friction between the sheet pile wall and the soil.

Application of the interface elements allows to model the interaction of structure and soil, i.e., the structure and the soil are bound together, and relative displacement is impossible without an interface. The behavior at the interface is controlled by the *R*, which determines the relationship between *φ* and the interface’s friction angle (*δ*’). It is assumed to be in the range of 0.5–0.8 between wall and soil [47]. Note that *R*_*inter*_ represents the *R* in the Plaxis program, and it is assumed that *R*_*inter*_ = 0.67 throughout the study as it is generally considered in practice. The relationship between *δ*’ and *φ*’ is as in Eq (1).


tanδ'=Rtan(φ') (1)

**Fig 2:**
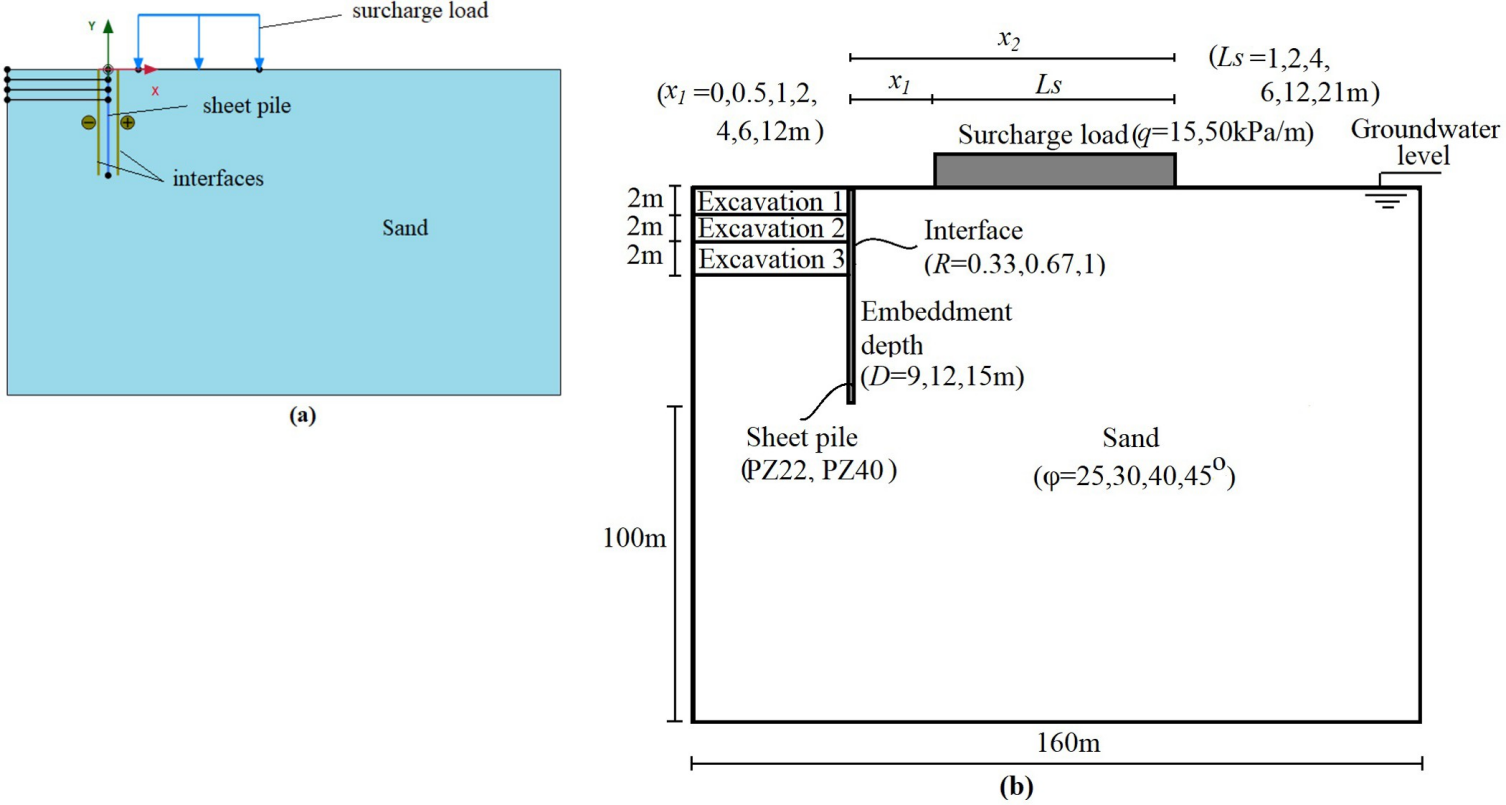
a) Simulation of the model considered in Plaxis 2D v20 and of which b) the scheme.

However, it is considered as 0.33, 0.67, and 1 in the study where the impact of the interface on the behavior of the wall is investigated. The groundwater table is assumed to be located at the natural ground surface for both the front and back of the wall. Staged construction is used as the loading type, and static stress calculations for the initial conditions are made in the first step. Then, the surcharge load and sheet pile are activated in the following phases. Finally, six meters of excavation, two meters at a stage, is finished in three steps. The developed model in the Plaxis 2D v20 program and the scheme that shows the model geometry and key variables are presented in Fig 2. At the same time, the material properties of the soil and the sheet pile wall are tabulated in Table 2, where E, A, I, EA, and EI are Young’s modulus, area, moment of inertia, the axial and flexural rigidity, respectively. Note that the properties of the PZ22 and PZ40 types of sheet piles are adopted from Amer [57].

**Table 2:** The properties of soil and the sheet pile materials.

Parameter	Unit	Soil	Sheet Piles	
		Sand	PZ22	PZ40
Model Material	-	Mohr-Coulomb	-	-
Material Type	-	Drained	Elastic	Elastic
*γ*_*s*_	kN/m^3^	21.0	-	-
*γ*_*n*_	kN/m^3^	19.0	-	-
*E*’	kN/m^2^	12000	-	-
*v*’	-	0.3	0.15	0.15
*c*	kN/m^2^	0.1	-	-
*EA*	kN/m	-	2.738E6	4.980E6
*EI*	kNm^2^/m	-	23.00E3	134.0E3

### Analytical methods

Today’s earth pressure calculations are generally based on Coulomb’s and Rankine’s theories. The primary difference between Rankine and Coulomb earth pressure theories is that Coulomb considers friction between the soil and wall [2]. Let’s consider a retaining wall with a back face inclined at *β* = 90° angle and a backfill made of granular soil that slopes at *α* = 0° angle to the horizontal to illustrate Coulomb’s active earth pressure theory. The active and passive lateral earth pressure coefficients can be calculated using Coulomb’s and Rankine’s theories as follows.

$$K_{a}=\frac{\sin^{2}\left(\beta +\varphi '\right)}{\sin^{2}\;\beta \sin\left(\beta -\delta '\right){\left[1+\sqrt{\frac{\sin\left(\varphi '+\delta '\right)\sin\left(\varphi '-\alpha \right)}{\sin\left(\beta -\delta '\right)\sin\left(\alpha +\beta \right)}}\right]}^{2}}$$ (2a)

$$K_{p}=\frac{\sin^{2}\left(\beta -\varphi '\right)}{\sin^{2}\;\beta \sin\left(\beta +\delta '\right){\left[1-\sqrt{\frac{\sin\left(\varphi '+\delta '\right)\sin\left(\varphi '+\alpha \right)}{\sin\left(\beta +\delta '\right)\sin\left(\alpha +\beta \right)}}\right]}^{2}}$$ (2b)

$$K_{a}=\tan^{2}\left(45-\frac{\varphi '}{2}\right)$$ (3a)

$$K_{p}=\tan^{2}\left(45+\frac{\varphi '}{2}\right)$$ (3b)

where *Ka* and *Kp* are the active and passive lateral earth pressure coefficients, respectively.

**Fig 3:**
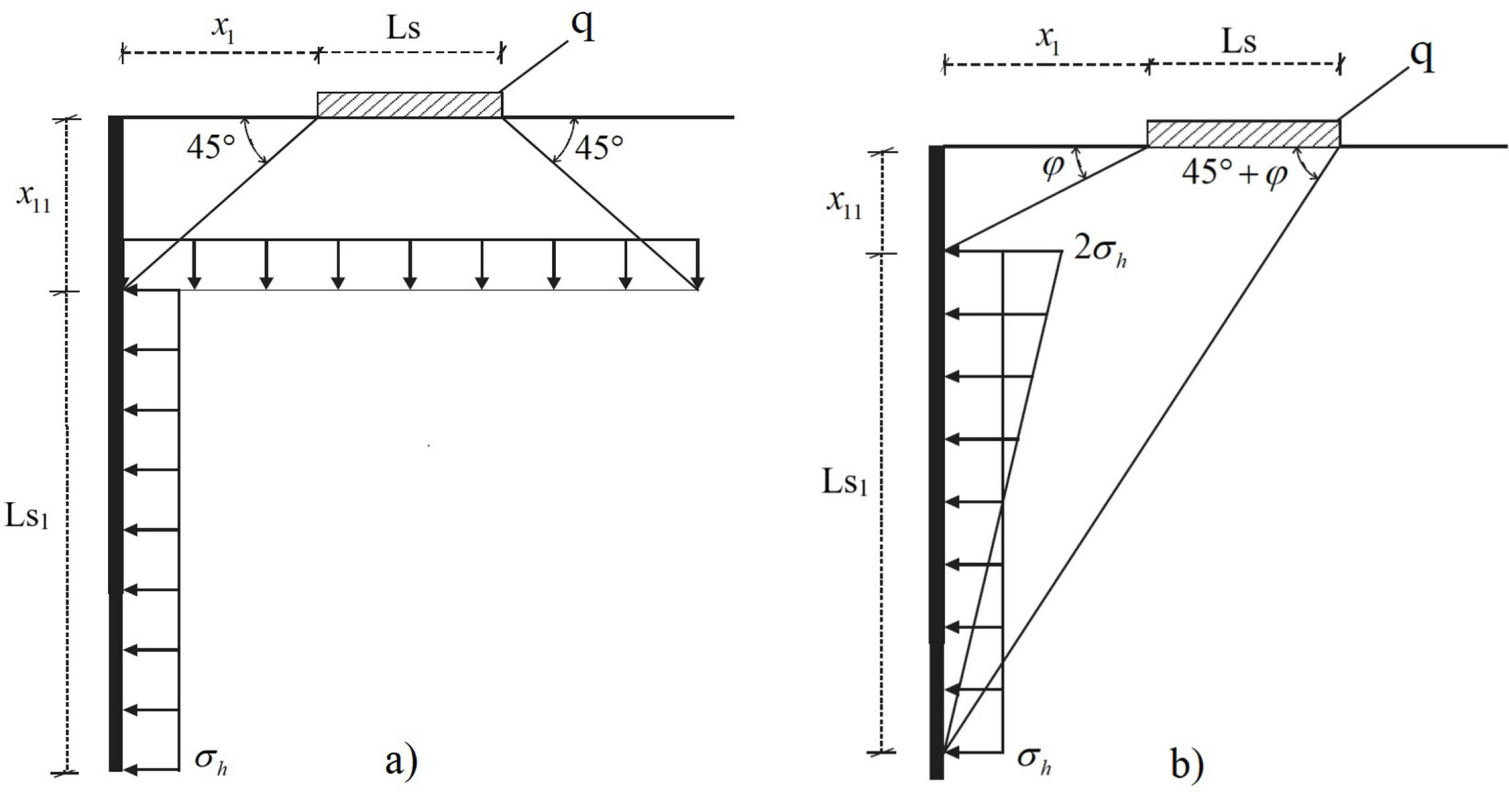
Approximate approaches: a) *AP* [19]; b) *BK* distribution [20, 58].

In the study, *AP* and *BK* methods are considered as load distribution approaches. Cernica (1995) [19] put forth the *AP* method, which distributes the strip load at a 45-degree angle (Fig 3A) while the strip load spreads at an angle between *φ’* and 45+*φ’* in *BK* [20] method (Fig 3B).

The *AP* approach is frequently used in practice due to its simplicity, and the lateral force caused by the surcharge load may be derived as follows.

$$\sigma_{h}=K_{a}\cos\delta '\frac{Ls}{Ls+2x_{1}}q$$ 4

$$Ls_{1}=Ls+2x_{1}\leq H+Z-x_{1}{}_{1}$$ (5)

$$x_{11}=x_{1}$$ (6)

$$Ps=qLsK_{a}$$ (7)

where *x*_11_, *Ls*_1_ and *Ps* are the depth, length, and resultant force of the lateral pressures caused by the surcharge load, respectively.

According to Beton-Kalender [20], the lateral force caused by the surcharge load can be determined as below.


$$\sigma_{h}=K_{a}\frac{qLs\cos\delta '\sin\left(45-\varphi /2\right)}{Ls_{1}\cos\left(45-\varphi /2-\delta \right)}$$ (8)

$$Ls_{1}=\left(x_{1}+Ls\right)\tan\left(45+\varphi '\right)-x_{1}\tan\varphi '$$ (9)

$$x_{11}=x_{1}\tan\varphi '$$ (10)

$$Ps=\sigma_{h}Ls_{1}$$ (11)

**Fig 4:**
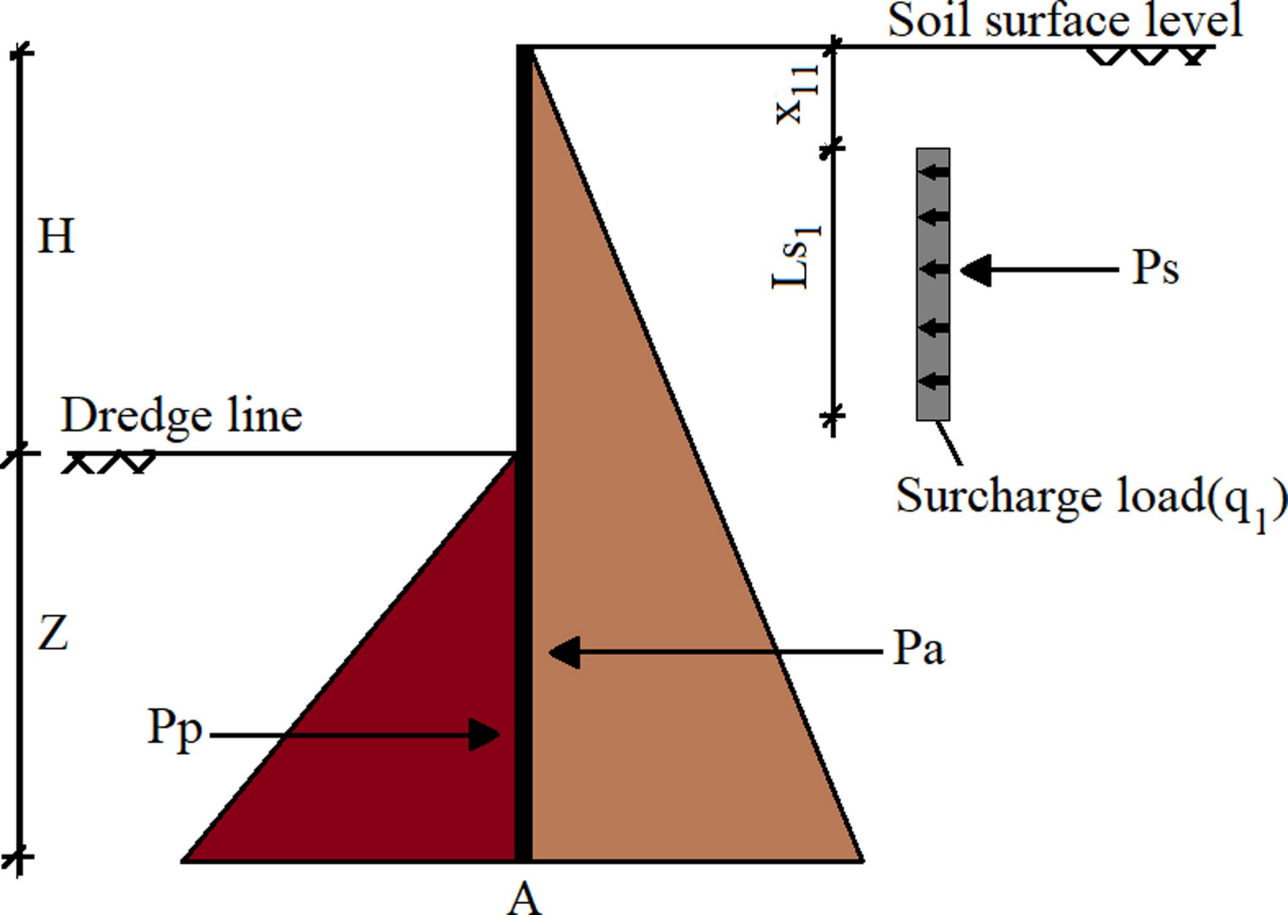
The lateral forces acting on sheet pile wall.

Note that it is assumed to be $\delta '=\frac{2}{3}\varphi '$ in Coulomb theory, while it is neglected in Rankine theory, i.e. δ’ = 0. Furthermore, the active and passive lateral earth pressures acting on the sheet pile wall are shown in Fig 4.

Hydrostatic pressures are not included in the calculations because the water levels are the same on both sides of the wall. Effective earth pressures are considered since the soil is fully saturated. The unit weight of water is assumed to be *γ*_*w*_ = 9.81 kN/m^3^, and the effective unit weight of the soil (*γ*^*’*^) is determined below.


$$\gamma '=\gamma_{s}-\gamma_{w}$$ (12)

Point A is located at a depth (*Z*) where the shear force is zero on the section of the sheet pile wall. Besides, *Pa* and *Pp* are the lateral resultant components of the active and passive forces until point A, respectively. Furthermore, *Pa* and *Pp* can be written as a function of *Z* as follows.


$$Pa=\frac{1}{2}K_{a}\gamma '{\left(H+Z\right)}^{2}\cos\delta '$$ (13)

$$Pp=\frac{1}{2}K_{p}\gamma 'Z^{2}\cos\delta '$$ (14)

It should be noted that each resultant force is inclined at an angle *α* with the horizontal in Rankine’s theory, while they are inclined at an angle *δ*’ with the normal of the wall in Coulomb’s theory. Therefore, the lateral components of these forces should be considered in calculations. After, the depth of *Z* is derived by using the balance of the total lateral force balance that is shown below:

$$\sum F_{x}=Pa+Ps-Pp=0$$ 15

*Pa*, *Pp*, and *Ps* act the wall at a point that is (*H*+*Z*)/3, *Z*/3 and $\left(H+Z-x_{1}-\frac{Ls_{1}}{2}\right)$ far from the base of the wall, respectively. Consequently, *Mmax* is determined by calculating the bending moment about the point A by the Eq (16).


$$M\max=Pa\frac{\left(H+Z\right)}{3}+Ps\left(H+Z-x_{1}-\frac{Ls_{1}}{2}\right)-Pp\frac{Z}{3}$$ 16

## Results and discussion

In this section, the findings of the current research are tabulated and illustrated regarding *Mmax* and *Ux* for various *q*, *x*_1_, *Ls*, φ, *D* values, and *ST*s of the sheet pile wall. It is considered that φ = 35, *R* = 0.67, *D* = 15m, *q* = 50kN/m and the *ST* is PZ40 unless otherwise stated. The analytical approaches consider Coulomb’s earth pressure theory with *AP* and *BK* and Rankine’s earth pressure theory with *AP* and *BK*, denoted by *APC*, *BKC*, *APR*, and *BKR*, respectively.

### Comparison studies

Table 3 compares the *Mmax* values obtained from the FE, *BKR*, *BKC*, *APR*, and *APC* methods for various φ, *x*_1_ and *Ls* values. The influence of φ and *x*_1_ / *H* on *Mmax* is more pronounced in the *FEM* and *BK* methods than in the AP method and becomes more pronounced as *Ls* increases. *Ls* substantially affects Mmax more in the *FEM* method than in the *AP* and *BK* methods. As φ, *x*_1_ / *H*, and *Ls* rise, the effect of the used methods on *Mmax* also increases. With an increase in φ and *x*_1_ / *H*, the influence of *Ls* on *Mmax* reduces, but *Ls* does not affect *Mmax* for large values of *x*_1_. *Mmax* values that account for Rankine earth pressures are higher than those that account for Coulomb earth pressures.

**Table 3:** *Mmax* values versus *x*_1_ / *H*, φ and *Ls*.

*Mmax*(kNm)											
*x*_1_ / *H*	φ (^ο^)	*Ls*(m)									
		1					2				
		*FE*	*APR*	*BKR*	*APC*	*BKC*	*FE*	*APR*	*BKR*	*APC*	*BKC*
0	25	-	660.91	660.91	422.07	422.07	770.50	843.96	843.96	548.84	548.84
	35	286.30	311.47	311.47	201.57	201.57	383.80	406.45	406.45	266.26	266.26
	45	157.00	159.08	159.08	105.01	105.01	197.80	209.86	209.86	139.58	139.58
0.33	25	-	579.74	623.06	352.99	389.86	650.20	681.62	768.26	410.68	484.42
	35	236.50	257.27	273.52	156.72	170.17	265.40	298.06	330.55	177.47	203.46
	45	106.80	124.77	124.77	77.23	77.23	110.40	141.64	141.64	86.36	86.36
0.67	25	-	476.58	547.36	285.82	326.00	-	488.58	616.85	289.66	360.65
	35	135.10	208.04	217.36	127.37	131.94	130.20	210.36	227.38	127.84	136.16
	45	69.15	101.22	101.22	63.74	63.74	63.09	101.67	101.67	63.78	63.78
1	25	-	463.16	480.45	281.48	287.71	284.40	463.16	495.81	281.48	293.17
	35	136.70	205.41	205.41	126.84	126.84	136.30	205.41	205.41	126.84	126.84
	45	76.59	100.71	100.71	63.71	63.71	67.55	100.71	100.71	63.71	63.71
2	25	-	463.16	463.16	281.48	281.48	-	463.16	463.16	281.48	281.48
	35	137.80	205.41	205.41	126.84	126.84	134.20	205.41	205.41	126.84	126.84
	45	70.50	100.71	100.71	63.71	63.71	69.32	100.71	100.71	63.71	63.71
		*Ls*(m)									
		4					6				
0	25	-	1164.33	1164.33	759.88	759.88	-	1421.73	1421.73	912.94	912.94
	35	451.20	562.41	562.41	365.15	365.15	473.20	672.09	672.09	422.93	422.93
	45	218.80	288.32	288.32	188.44	188.44	229.10	335.61	335.61	210.15	210.15
0.33	25	879.50	1001.99	1088.63	621.72	695.46	930.10	1178.22	1308.18	705.70	816.30
	35	365.40	454.01	486.51	275.46	302.35	362.10	509.49	558.24	296.47	329.69
	45	163.80	219.69	219.69	132.94	132.94	151.10	237.27	237.27	141.83	141.83
0.67	25	762.60	839.65	1012.93	486.02	631.03	809.40	936.10	1194.63	531.75	719.67
	35	286.70	349.98	410.61	204.11	241.27	285.60	382.01	449.66	220.52	260.70
	45	101.70	163.29	163.29	97.91	97.91	98.83	176.65	176.65	104.91	104.91
1	25	550.00	602.01	861.52	345.08	502.68	558.80	646.66	967.53	364.92	550.18
	35	172.20	248.58	295.60	145.61	172.25	164.50	261.94	319.47	151.22	183.82
	45	66.17	116.38	116.38	70.38	70.38	65.32	121.10	121.10	72.32	72.32
2	25	393.20	509.12	717.62	296.13	410.87	378.00	526.01	787.94	301.38	445.39
	35	135.50	214.27	243.22	128.61	142.70	126.30	217.43	255.15	129.23	147.55
	45	61.55	102.42	102.42	63.83	63.83	59.28	103.01	103.01	63.87	63.87
		*Ls*(m)									
		12					*∞*				
0	25	-	1800.29	1800.29	1041.01	1041.01	-	1805.06	1805.06	1041.01	1041.01
	35	471.10	750.43	750.43	439.45	439.45	461.90	750.43	750.43	439.45	439.45
	45	236.90	351.39	351.39	211.35	211.35	236.20	351.39	351.39	211.35	211.35
0.33	25	955.70	1370.29	1579.77	778.53	904.89	946.20	1541.66	1678.26	867.37	956.98
	35	379.00	559.27	608.45	322.64	352.62	375.10	621.66	658.29	355.12	378.91
	45	148.70	258.50	258.50	153.32	153.32	171.10	284.89	284.89	167.41	167.41
0.67	25	795.50	1077.94	1394.33	603.36	793.01	830.30	1309.38	1558.31	717.75	878.25
	35	297.20	431.96	502.21	245.69	288.00	298.70	511.35	575.28	284.75	325.28
	45	92.05	197.20	197.20	115.49	115.49	97.01	229.19	229.19	131.59	131.59
1	25	549.80	732.90	1111.25	402.55	623.20	552.90	934.33	1338.57	487.57	736.37
	35	176.60	287.17	360.30	161.62	203.24	178.70	343.73	435.84	184.08	238.12
	45	67.24	129.85	129.85	75.86	75.86	61.59	148.83	148.83	83.24	83.24
2	25	387.10	562.32	910.76	312.47	504.67	376.90	672.50	1145.12	344.75	614.81
	35	129.50	224.06	277.99	130.50	156.64	127.50	243.18	330.56	133.98	176.77
	45	59.87	104.24	104.24	63.96	63.96	59.43	107.64	107.64	64.18	64.18

**Fig 5:**
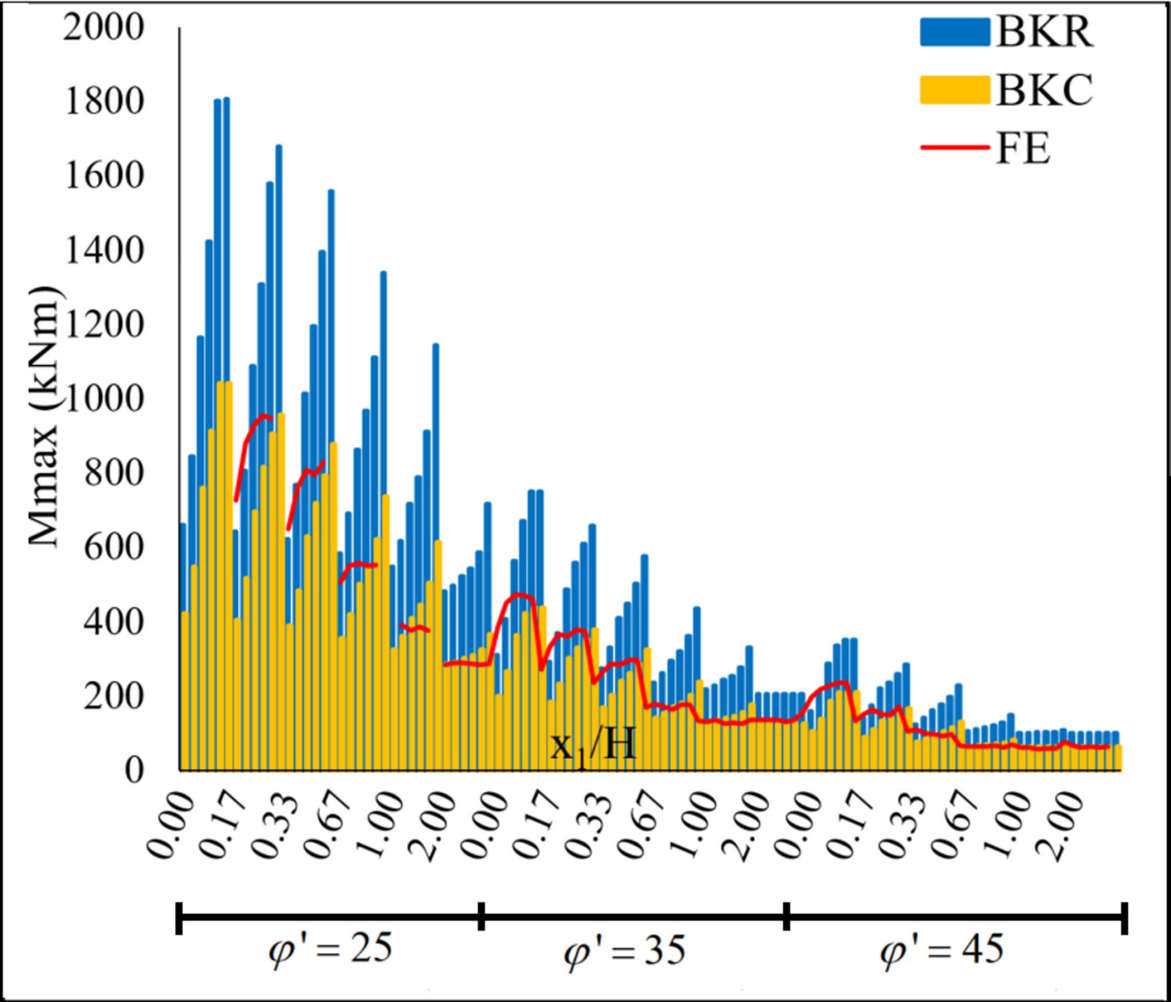
The variation of *Mmax* values versus *φ*, *Ls* and *x*_1_ / *H*.

The change in *Mmax* values obtained from the FE, *BKR*, and *BKC* methods versus φ and *x*_1_ is shown in Fig 5. *Mmax* results obtained from the *BKR* method are significantly higher, while *Mmax* results obtained from the *BKC* method are slightly lower than those for *FEM*. The difference between the results obtained by *FEM* and *BKC* methods becomes more pronounced while *x*_1_ / *H* and *Ls* decrease.

**Fig 6:**
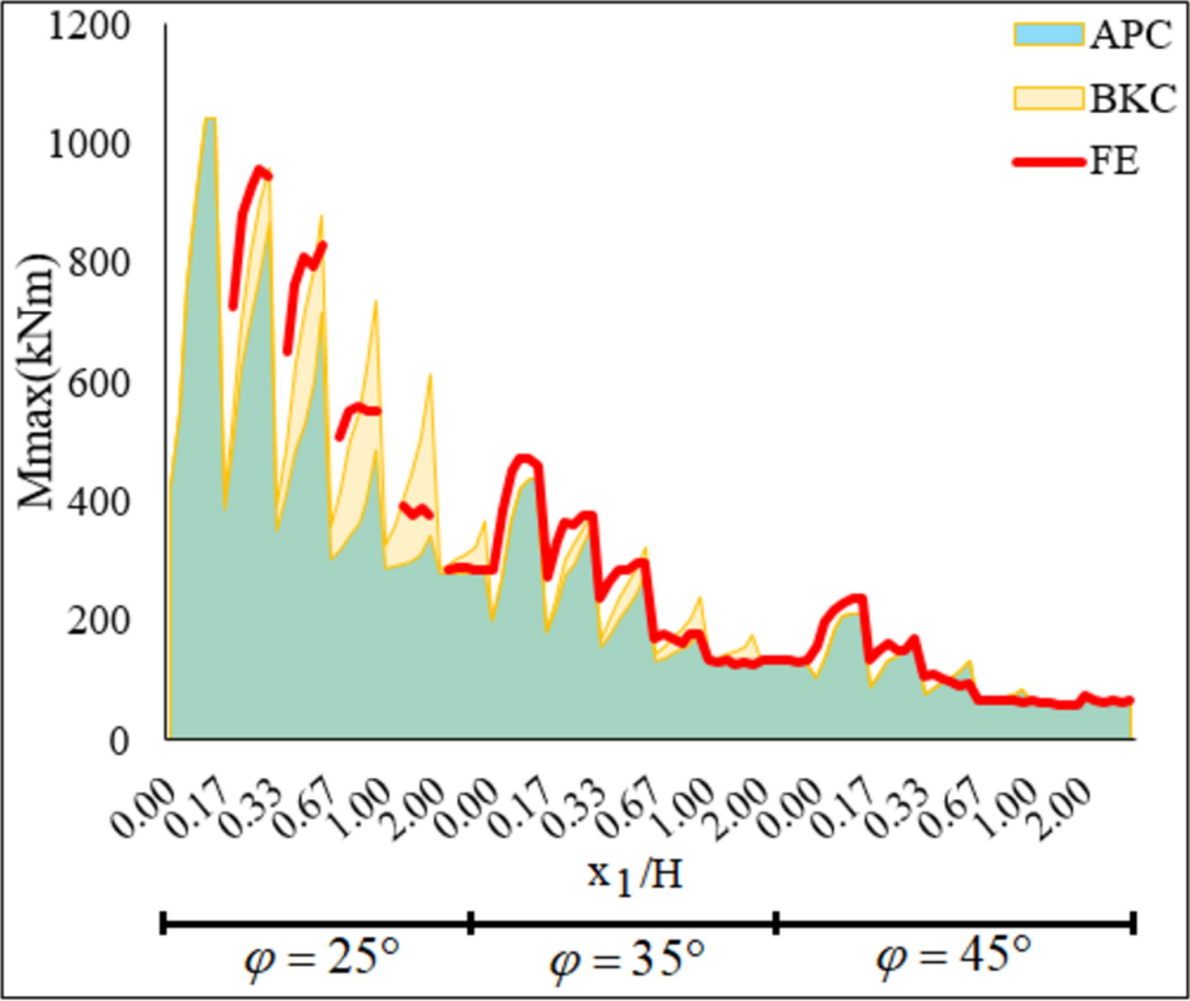
The variation of *Mmax* values versus *φ*, *Ls* and *x*_1_ / *H*.

The change of *Mmax* values found using the FE, *BKC*, and *APC* methods regarding the φ and *x*_1_ / *H* is shown in Fig 6. *Mmax* values obtained from the *BKC* method appear to be higher than those acquired using the *APC* method. The effect of the load distribution approaches on *Mmax* becomes more evident as the *x*_1_ / *H* increase and the φ decrease. The *Mmax* values obtained from *FEM* are generally less than those from *BKC*, and the discrepancy gets more significant as *x*_1_ rises. However, the results obtained using *FEM* are greater than those from *APC*. Therefore, it is more conservative to use the BK method in analytical solutions. Consequently, the evaluations show that the results of the numerical and analytical solutions are consistent. This demonstrates the worth of the FEM results. Furthermore, FEM is used for parametric study.

### Parametric studies

The effects of *D*, *R*, *Ls*, *q*, *x*_1_, and *ST* on the *Mmax* and *Ux* are investigated using *FEM* within the parametric studies. *D* is anticipated to have minimal effect, whereas the others should have a significant effect.

#### Study 1

Table 4 presents the variation of *Mmax* values versus *D/H* and *q* for different *φ* and *Ls* values where *x*_1_ = 0. *Mmax* varies slightly while *D/H* increases, so that indicates that *D/H* does not influence *Mmax*. Additionally, *Mmax* increases linearly while *q* rises regardless of *Ls* and φ. *Mmax* reduces roughly as φ increases from 30 to 45 degrees.

**Table 4:** *Mmax* values versus *φ*, *q*, *D*, and *Ls*.

*D/H*	*q*(kN/m)	*Ls*(m)	*Mmax*(kNm)			
			φ(^ο^)			
			30	35	40	45
1.5	0	0	188.40	134.40	93.49	67.83
	15	6	319.20	223.40	158.60	110.90
		21	336.00	236.10	167.30	115.60
		*∞*	-	224.50	155.50	110.30
	50	6	702.40	472.70	-	219.70
		21	717.60	473.10	348.30	235.50
		*∞*	695.80	463.50	327.50	227.00
2	0	0	191.90	139.70	99.75	71.31
	15	6	321.00	219.10	158.80	111.40
		21	330.80	229.80	165.40	116.90
		*∞*	334.60	224.00	163.80	114.70
	50	6	694.60	469.80	324.30	219.20
		21	691.70	484.00	342.70	239.10
		*∞*	695.80	463.80	335.00	230.10
2.5	0	0	189.20	135.80	95.46	67.06
	15	6	318.60	221.40	156.60	112.80
		21	332.10	232.30	167.90	116.20
		*∞*	328.60	227.40	162.50	114.00
	50	6	699.10	473.20	325.20	229.10
		21	-	482.10	344.40	233.50
		*∞*	711.00	466.80	334.10	232.70

#### Study 2

Table 5 demonstrates the variation of *Mmax* values with respect to the *R*, φ, *x*_1_ for various *Ls* values. *Mmax* increases while *R* decreases, and the effect of *R* on *Mmax* depends on the φ but is independent of *x*_1_ and *Ls*. Note that *Mmax* decreases where φ = 25 and *R =* 0.33 as an exception. It is predicted that the system deviates from stable conditions and experiences significant movements with bearing capacity losses due to the very loose soil and insufficient interface friction.

**Table 5:** *Mmax* values versus *φ*, *x*_1_ / *H*, and *R*.

*Mmax*(kNm)										
*x*_1_ / *H*	φ (^ο^)	*R =* 1	*R =* 0.67	*R =* 0.33	*R =* 1	*R =* 0.67	*R =* 0.33	*R =* 1	*R =* 0.67	*R =* 0.33
		*Ls*(m)								
		1			2			4		
0	25	-	-	-	-	770.50	-	897.90	-	-
	35	263.70	286.30	344.20	354.90	383.80	-	427.00	451.20	-
	45	146.50	157.00	185.30	188.80	197.80	254.20	215.20	218.80	274.50
0.17	25	-	-	-	670.20	727.10	-	802.50	879.50	-
	35	250.10	272.10	-	314.20	334.00	-	342.70	365.40	-
	45	127.80	134.20	-	152.60	150.80	-	152.90	163.80	-
0.33	25	-	-	417.90	596.20	650.20	509.90	709.70	762.60	558.90
	35	217.80	236.50	289.90	255.40	265.40	343.00	268.70	286.70	400.40
	45	98.39	106.80	132.30	104.50	110.40	133.10	103.20	101.70	124.40
0.67	25	396.90	-	-	458.50	507.20	-	-	550.00	-
	35	161.10	170.40	-	157.30	180.20	-	160.50	172.20	-
	45	63.67	67.82	-	64.94	65.86	-	67.34	66.17	-
1	25	-	-	-	-	-	252.90	-	393.20	256.30
	35	123.30	135.10	-	120.20	130.20	159.70	119.10	135.50	169.90
	45	62.86	69.15	85.50	60.38	63.09	85.14	61.74	61.55	81.89
2	25	260.50	-	-	260.40	284.40	-	259.90	289.30	-
	35	122.50	136.70	-	120.30	136.30	-	117.60	136.30	-
	45	58.87	76.59	-	59.45	67.55	-	57.15	62.02	-
		*Ls*(m)								
		6			12			*∞*		
0	25	-	-	879.40	-	-	-	983.60	-	844.70
	35	-	473.20	600.50	439.50	471.10	-	438.10	461.90	597.40
	45	210.80	229.10	263.00	206.20	236.90	271.70	205.20	236.20	281.00
0.17	25	844.00	930.10	-	853.40	955.70	-	863.10	946.20	-
	35	355.10	362.10	-	349.30	379.00	-	372.00	375.10	-
	45	139.10	151.10	-	147.20	148.70	-	146.30	171.10	-
0.33	25	733.40	809.40	568.90	740.00	795.50	568.10	732.70	830.30	574.00
	35	265.70	285.60	358.00	267.90	297.20	374.80	270.40	298.70	361.60
	45	107.70	98.83	127.00	103.30	92.05	126.20	92.89	97.01	138.30
0.67	25	496.30	558.80	-	-	549.80	-	494.30	552.90	-
	35	153.00	164.50	-	149.90	176.60	-	162.70	178.70	-
	45	69.75	65.32	-	72.91	67.24	-	61.64	61.59	-
1	25	-	378.00	252.10	352.20	387.10	250.80	352.10	376.90	258.90
	35	118.80	126.30	163.50	120.00	129.50	176.70	118.90	127.50	166.70
	45	60.19	59.28	78.06	63.95	59.87	76.22	56.66	59.43	83.96
2	25	260.30	289.10	-	259.90	287.30	-	259.80	285.00	-
	35	119.10	136.00	-	117.20	131.70	-	123.20	135.50	-
	45	58.67	65.38	-	57.33	62.38	-	58.05	65.34	-

#### Study 3

**Fig 7:**
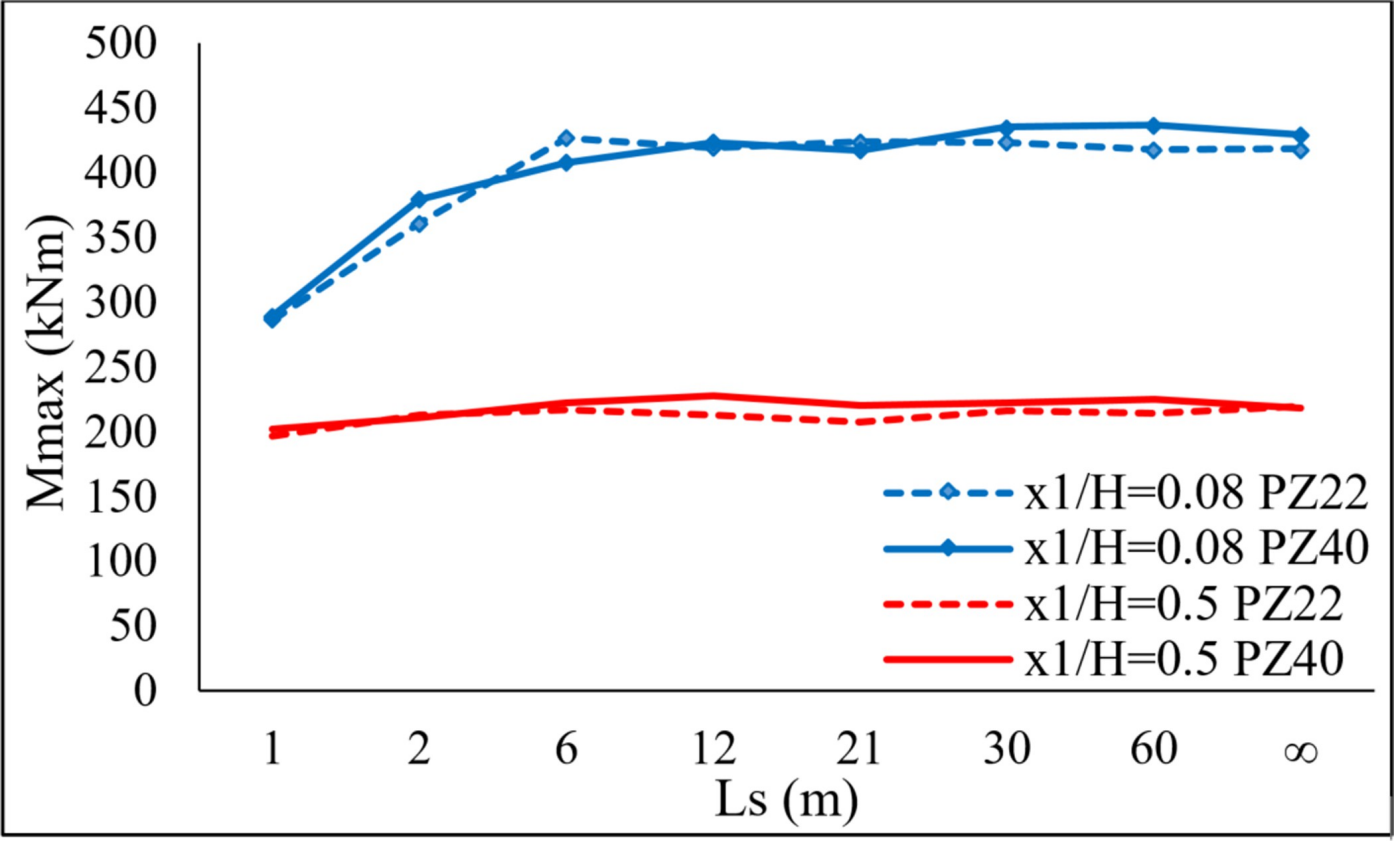
*Mmax* values versus *Ls* and the *ST*s of PZ22 and PZ40.

The variance of the *Mmax* values versus the *ST* of the sheet pile and *Ls* is shown in Fig 7. The *Mmax* values for PZ22 and PZ40 differ slightly, so the *ST* has a negligible impact on *Mmax*. The fluctuation of *Mmax* caused by *Ls* depends on *x*_1_ / *H* while it is independent of the *ST*.

#### Study 4

Table 6 exhibits the *Ux* and *Mmax* values depending on the distance of the endpoint of the surcharge load (*x*_2_) for *x*_1_ / *H* = 0.08 and *x*_1_ / *H* = 0.5. *Mmax* and *Ux* rise with the increase in *x*_2_ / *H* but once *x*_2_ / *H* exceeds a certain point, the effect on *Mmax* and *Ux* disappears.

**Table 6:** *Mmax* and *Ux* values versus *x*_2_ / *H*.

*x*_2_ / *H*	*Ux*(cm)	*Mmax*(kNm)	*Ux*(cm)	*Mmax*(kNm)
	*x*_1_ / *H*			
	0.08		0.5	
0.17	-6.60	224.10	-	-
0.33	-12.59	339.90	-	-
0.67	-18.66	429.60	-6.25	202.40
0.83	-19.07	420.50	-7.62	210.60
1.00	-19.66	425.40	-8.26	214.60
1.17	-19.63	421.30	-8.78	216.00
1.33	-19.79	426.40	-8.38	217.90
1.50	-	-	-9.02	222.10
1.67	-	-	-7.65	209.40
2.00	-18.59	420.00	-	-
2.50	-	-	-7.77	227.90
3.50	-18.64	429.00	-7.10	220.20
5.00	-17.87	425.70	-6.62	222.50
7.50	-	-	-6.28	224.40
10.00	-17.12	421.40	-6.02	225.00
*∞*	-17.23	429.30	-5.58	218.20

**Fig 8:**
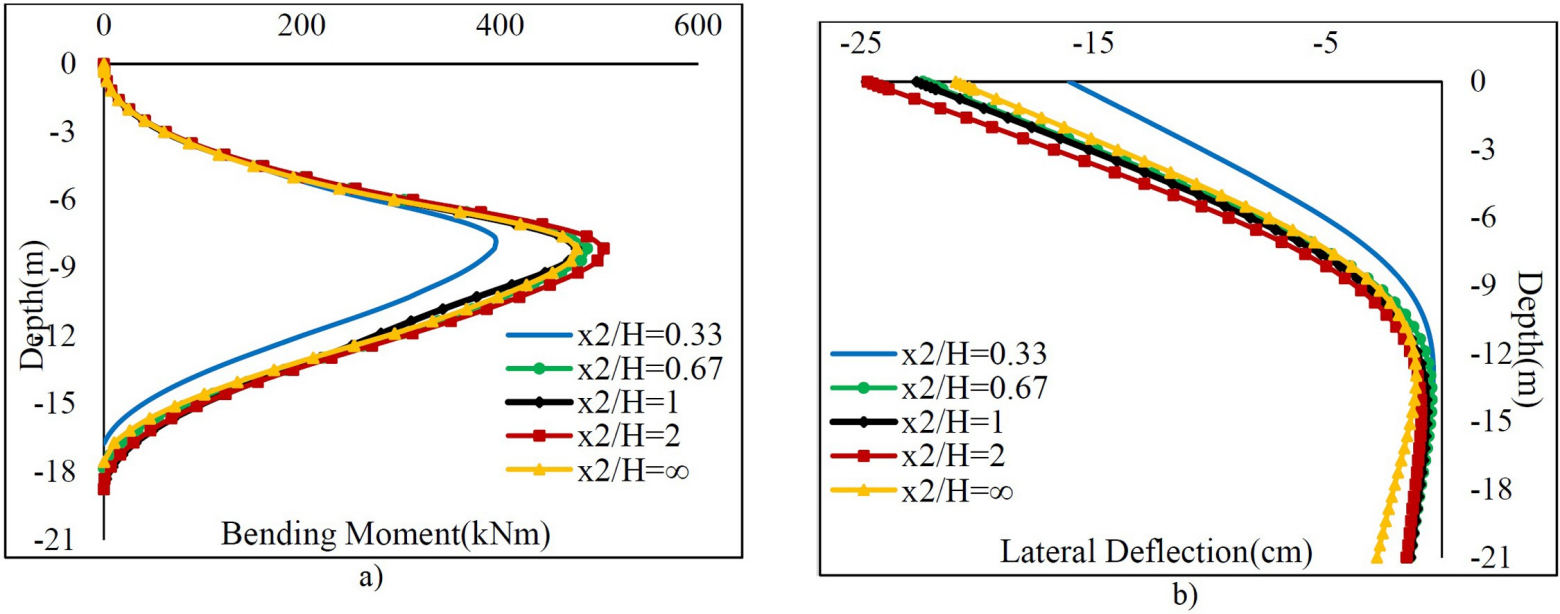
The variation of a) bending moment and b) lateral deflection along the sheet pile.

Fig 8 shows the variation of bending moments and lateral deflections along the sheet pile wall versus the *x*_2_ / *H* for *x*_1_ = 0. The *Mmax* values increase nonlinearly as the *x*_2_ / *H* rises but stop increasing after a certain point.

**Fig 9:**
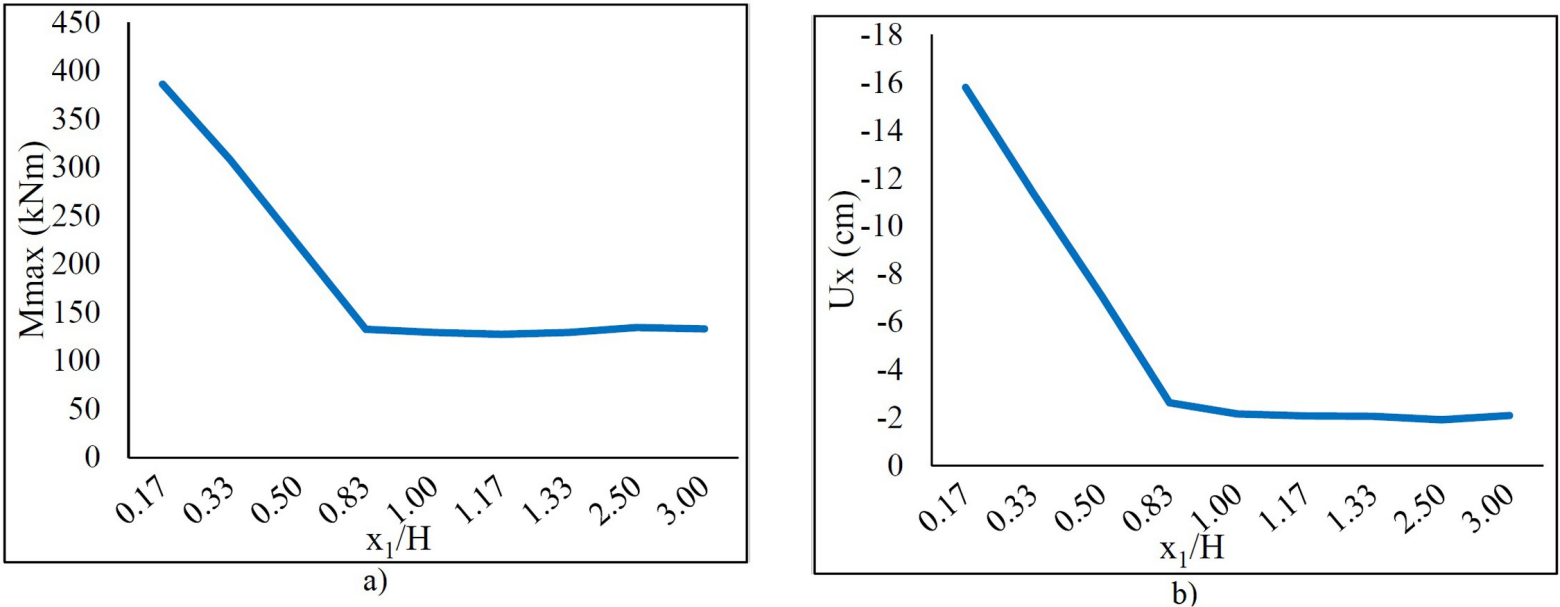
The variation of a) *Mmax* and b) *Ux* versus *x*_1_ / *H*.

Additionally, Fig 9 depicts the change of *Mmax* and *Ux* against the *x*_1_ where *x*_2_ / *H* = 3. *Mmax* and *Ux* decrease linearly with the increase in *x*_1_ but remain constant after *x*_1_ reaches larger values. Therefore, the surcharge load should be considered for the safety of the wall in case the surcharge is close to the wall.

#### Study 5

**Fig 10:**
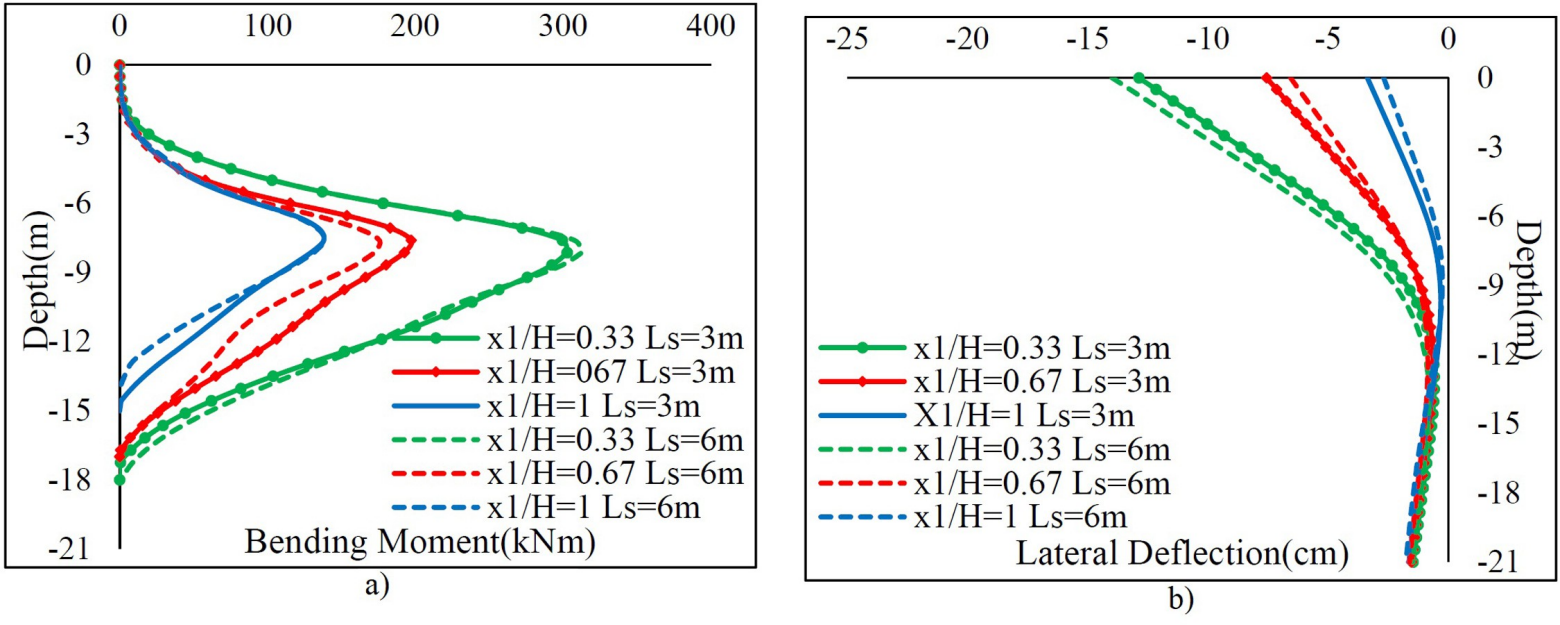
The variation of a) bending moment and b) lateral deflection along the sheet pile.

Table 7 demonstrates the fluctuation of *Mmax* and *Ux* values versus *x*_1_ / *H* for *Ls* = 3m and *Ls* = 6m. Additionally, Fig 10 illustrates the variation of bending moments and lateral deflections along the sheet pile wall depending on *x*_1_ values. As *x*_1_ / *H* decreases and *Ls* increases, both *Mmax* and *Ux* increase. Furthermore, it can be concluded that the effect of *x*_1_ / *H* on *Mmax* and *Ux* is more pronounced than that of *Ls*.

**Table 7:** The variation of *Mmax* and *Ux* versus *x*_1_ / *H*.

*x*_1_ / *H*	*Ux*(cm)	*Mmax*(kNm)	*Ux*(cm)	*Mmax*(kNm)
	*Ls*(m)			
	3		6	
0.08	-	-	-18.66	407.40
0.17	-15.63	372.80	-16.46	362.10
0.33	-11.26	282.00	-11.97	285.60
0.5	-8.26	214.60	-9.02	222.10
0.67	-6.21	166.90	-5.18	164.50
0.83	-4.67	143.40	-3.39	139.50
1	-3.06	132.50	-2.35	126.30
1.17	-2.16	129.70	-2.09	127.20
1.33	-	-	-	-
2.5	-2.22	135.70	-1.91	134.50
3	-2.09	133.10	-2.23	138.00
3.5	-2.02	131.90	-2.05	135.20
4.5	-2.14	133.80	-1.90	131.80
7	-2.45	138.00	-2.16	133.60

## Conclusions

The present study examines the effect of the surcharge load with various *x*_1_, *Ls*, *q*, *φ*, *R*, *D* values, and different *STs* on the *Mmax* and *Ux* using *FEM* analyses. Besides, four distinct analytical methods are used to solve the *Mmax* of the models combining Coulomb’s and Rankine’s earth pressure theories with *AP* and *BK* distribution approaches to allow one to compare the consistency of widely used analytical solutions with FEM. The following findings are stated briefly:

* *Mmax* values obtained from Rankine’s theory are higher than those for Coulomb’s theory.
* The *Mmax* values obtained from *FEM* are generally lower than those from *BKC*, while the results of *FEM* are greater than those of *APC*.
* The effect of the applied methods on *Mmax* becomes more evident for a higher distance of the surcharge load and lower internal friction angle of the soil.
* The embedment depth and section type of the sheet pile do not influence *Mmax* significantly.
* *Mmax* increases linearly while the intensity of the surcharge load rises.
* *Mmax* increases as internal and interface friction angle decreases, with some exceptions where the sand is very loose and the interface friction is very low.
* *Mmax* and *Ux* increase as both the surcharge load approaches the wall and the length of the surcharge load increases.
* The effect of the distance of the surcharge load is more pronounced than that of the length.
